# Current research status and clinical applications of noninvasive preimplantation genetic testing: A review

**DOI:** 10.1097/MD.0000000000039964

**Published:** 2024-10-04

**Authors:** Shaozhe Yang, Bo Xu, Yuan Zhuang, Qingwei Zhang, Junfeng Li, Xiuhong Fu

**Affiliations:** a Henan Key Laboratory of Fertility Protection and Aristogenesis, Luohe Central Hospital, Luohe, People’s Republic of China; b Luohe Reproductive Medicine and Genetics Center, Luohe Central Hospital, Luohe, People’s Republic of China.

**Keywords:** blastocoel fluid, cell-free DNA, embryo culture medium, noninvasive preimplantation genetic testing

## Abstract

Noninvasive preimplantation genetic testing (ni-PGT) is conducted by obtaining genetic information from embryos through the analysis of free DNA released by embryos in spent embryo culture medium or blastocoel fluid. Compared to conventional preimplantation genetic testing relying on trophectoderm biopsy, ni-PGT is characterized by its noninvasiveness. It has demonstrated early advancements in the detection of embryonic chromosomal aneuploidies and the diagnosis of monogenic diseases, showcasing considerable potential for clinical application. However, there are substantial controversies in the literature concerning the reliability of ni-PGT, the source of cell-free DNA, and maternal contamination. This paper elaborates on the principles, research advancements, effectiveness, and limitations of ni-PGT to provide a basis for clinical applications.

## 1. Introduction

Birth defects are a major cause of early miscarriage, stillbirth, infant mortality, and congenital disabilities.^[1]^ In the United States, 20.6% of infant deaths are caused by birth defects,^[2]^ with 80% of these Birth defects being directly or indirectly caused by genetic factors.^[3]^ The report indicates that chromosomal aneuploidy and polyploidy were detected in 50% of early miscarriage embryonic tissues.^[4,5]^ Pathogenic copy number variations are also closely related to early miscarriages.^[6,7]^ Early natural miscarriages are an important factor affecting the success rate of assisted reproductive technology (ART).^[8]^ The rate of occurrence of chromosome aneuploidy in embryos increases with the age of pregnant women,^[9]^ leading to a significantly higher rate of ART failure and birth defects in older women compared to younger women.^[10]^ The noninvasive, simple, and accurate selection of high-quality embryos with optimal developmental potential before embryo transfer is a focal point of research in the fields of ART and genetics.

Preimplantation genetic testing (PGT) involves the biopsy of embryos before transfer, followed by genetic testing to select healthy embryos for transfer in ART. This technique significantly reduces the occurrence of fetal chromosomal abnormalities, recurrent miscarriages, and hereditary genetic diseases.^[11,12]^ Since the initial report by Handyside et al,^[13]^ demonstrating the use of PCR to select male embryos by detecting Y chromosome-specific repeat sequences to prevent the transmission of X-linked adrenoleukodystrophy, the applications of PGT have continuously expanded. These applications range from monogenic diseases (preimplantation genetic testing for monogenic diseases)^[14]^ to chromosomal aneuploidies (preimplantation genetic testing for aneuploidy [PGT-A])^[15]^ and structural abnormalities such as translocations and inversions (preimplantation genetic testing for structural rearrangements).^[16]^

Lo et al^[17]^ discovered cell-free fetal DNA in maternal peripheral blood of pregnant women, leading to the development of noninvasive prenatal testing. Preimplantation embryos may also release cell-free DNA (cfDNA) into the embryo culture medium, enabling noninvasive preimplantation genetic screening or diagnosis of embryos. Palini et al^[18]^ and Stigliani et al^[19]^ successively detected cfDNA in blastocoel fluid (BF) and spent embryo culture medium (SCM), marking the inception of ni-PGT for embryos.

Despite the considerable research conducted by many scholars on the sources and reliability of cfDNA in ni-PGT, there is a wide disparity in the conclusions drawn. This article provides a comprehensive review of recent advancements, limitations, potential applications, and challenges in ni-PGT research, offering insights for the clinical implementation of ni-PGT.

## 2. The limitations of traditional PGT

The global utilization of PGT in ART is on the rise. According to the latest report by the European Society of Human Reproduction and Embryology (2022), in 2018, 4.8% of ART cycles in Europe involved PGT. In the United States, the proportion of ART cycles involving PGT increased from 5% to 22% between 2015 and 2016.^[20]^ The limitations of preimplantation genetic testing (PGT) primarily manifest in the potential damage to embryos during biopsy procedures, the possible misdiagnosis of mosaic embryos, and the high level of technical expertise required.

### 2.1. Potential damage to embryos during biopsy

Regardless of preimplantation genetic testing for monogenic diseases, PGT-A, or preimplantation genetic testing for structural rearrangements, biopsy of the blastomere or trophectoderm (TE) is required.^[21]^ Currently, most in vitro fertilization (IVF) laboratories typically perform embryo biopsy by laser hole drilling or mechanical dissection to extract 1 or more embryonic cells,^[22]^ which is an invasive microsurgical procedure.^[23]^ The potential long-term effects of this method on embryo development and individual health remain to be further validated.^[15]^ Laser drilling or mechanical dissection of embryos may result in chromosomal loss.^[24]^ Kirkegaard et al^[25]^ observed, through time-lapse imaging, that embryos subjected to PGT required a longer time to develop into blastocysts compared to blastocysts without PGT intervention. Animal experiments have shown that mice born after blastomere biopsy exhibit impaired adrenal gland or neural tube development,^[26–28]^ and an increased risk of neurodegenerative diseases.^[29]^ Zhang et al^[30]^ reported a threefold increase in the incidence of preeclampsia in pregnant women undergoing TE biopsy for PGT, suggesting a potential impact of TE biopsy on embryo quality and fetal development. Notably, PGT technology was developed in 1990.^[13]^ With <35 years of application, it is challenging to evaluate the health status of individuals born through PGT in their middle or old age.

### 2.2. Ineffective diagnosis of mosaic embryos

PGT assesses the genetic information of a whole embryo (WB) based on 1 to 2 cells (blastomeres) or 5 to 10 cells (TE cells). The presence of embryo mosaicism can compromise accuracy and lead to potential misdiagnoses in PGT. In fact, human embryo mosaicism occurs at a notable rate. The latest single-cell sequencing results reveal that at least 80% of human embryos exhibit mosaic aneuploidy.^[31]^ It is possible that the findings of PGT do not accurately represent the chromosomal status of the WB or the eventual fetal development.^[32,33]^ There may be inconsistencies between the TE chromosomes and the inner cell mass (ICM), with the TE representing the lineage for placental development, whereas the ICM gives rise to the fetus. Research indicates the presence of self-correction capabilities in embryos, where aneuploid cells produced during embryonic development are preferentially assigned to the TE rather than the ICM.^[34]^ Malvestiti et al^[35]^ reported 60,347 cases of chorionic villus sampling, among which 1317 were classified as mosaics. Only 13% of these cases were verified as authentic fetal mosaicism, providing additional evidence of potential chromosomal disparities between the placenta/TE and the fetus/ICM.

### 2.3. The high level of technical expertise in PGT

The implementation of PGT not only requires expensive equipment in IVF and genetic laboratories, but also demands skilled embryologists to perform intricate procedures. This raises the cost of ART and hinders the widespread adoption of PGT. Up to the end of 2023, there are 454 reproductive medicine centers authorized to provide IVF and intracytoplasmic sperm injection (ICSI) techniques in China, yet only 110 of them are approved for PGD services. This limitation restricts access to PGT, particularly for the growing population of older individuals experiencing infertility who could benefit from this technology. The potential widespread application of ni-PGT could enable more IVF centers and patients to engage in PGT of embryos. The quest for noninvasive, safe, and accurate methods for chromosomal screening of embryos and assessment of embryo quality has garnered significant attention from reproductive medical professionals, emerging as a focal point and challenge in current clinical research.

## 3. The discovery of cfDNA in BF and SCM

Katz-Jaffe et al^[36]^ conducted a proteomic study on interleukins, proteins, and other metabolites in SCM, revealing a correlation between the levels of various metabolites in SCM and the implantation potential of embryos. However, they did not investigate cfDNA, which may have the closest association with embryo aneuploidy.

Currently, there are 2 approaches for cfDNA sampling in ni-PGT: 1 involves obtaining BF through blastocyst micropuncture and aspiration of BF,^[37]^ while the other entails using SCM that have been previously used to culture blastomeres or culture blastocysts. BF sampling typically employs an ICSI needle to penetrate the opposite side of the blastocyst ICM and extract fluid, resulting in complete embryo collapse,^[37]^ similar to the removal of BF during blastocyst cryopreservation to prevent ice crystal formation. Research indicates that the loss of BF does not affect embryo development or viability.^[38]^ The use of SCM for detection does not require a puncture operation.

Stigliani et al^[19]^ used PCR amplification to identify genomic DNA and mitochondrial DNA in the SCM of 800 embryos. Out of the total, 326 SCM samples were found to contain either genomic DNA or mitochondrial DNA (205 cases had genomic DNA; 322 cases had mitochondrial DNA). This study confirmed the presence of cfDNA in SCM for the first time. In the same year, Palini et al^[18]^ employed fluorescence quantitative PCR to analyze BF collected during the vitrification process of embryos, detecting the TBC1D3 gene on chromosome 17 and the TSPY1 gene on the Y chromosome to screen male embryos. The results showed that genomic DNA was found in 90% of BF. This study marks the initial observation of detecting embryonic cfDNA in BF.

## 4. The origins of cfDNA

Clarifying the origin of cfDNA and its generation mechanisms serves as the theoretical basis for utilizing cfDNA in ni-PGT. A key question is whether cfDNA is produced by embryos under normal physiological conditions or in pathological states, and whether aneuploid and euploid cells release comparable amounts of cfDNA. Currently, there is insufficient evidence to ascertain the sources and biological mechanisms of cfDNA. Further research is needed to determine the precise origins of cfDNA and the extent to which DNA from each source independently reflects the genetic status of the embryo.

In noninvasive prenatal testing, cfDNA from apoptotic placental cells is used for analysis. Similarly, in the field of ni-PGT, cfDNA may be produced through similar processes. Zhang et al^[39]^ performed sequencing on BF-cfDNA and found 2 distinct populations of DNA fragment sizes. The first peak included fragments ranging from 160 to 220 bp, with a prominent peak at 169 bp, similar to apoptotic fragments in human plasma. The second peak was broader, with fragment sizes from 300 to 400 bp, possibly from cellular necrosis. Studies have also compared cfDNA levels in culture media with and without embryos, showing higher cfDNA detection in media with embryos.^[40]^ These results suggest that cfDNA in culture media may come from embryonic release.

Researchers have proposed several hypotheses regarding whether cfDNA is produced by embryos under normal physiological conditions or in pathological states, suggesting that cfDNA in culture media may originate from apoptotic or necrotic cells during embryo development,^[40–42]^ or from cell damage caused by laser pulses used in artificial shrinkage of blastocysts,^[43]^ or from processes such as cell mitosis.^[44]^ cfDNA may also originate from active release during embryonic growth, as there have been reports of extracellular vesicles containing DNA being detectable in SCM.^[45]^ Some scholars believe that cfDNA arises from self-correction mechanisms during embryo development, where abnormal cells, such as those with chromosomal aneuploidies generated during cell division, are “discarded” by the embryo.^[46,47]^ However, Vera-Rodriguez et al^[40]^ found that the concentration of SCM-cfDNA is not related to the presence of aneuploid embryos by testing SCM-cfDNA from both euploid and aneuploid embryos. Overall, most researchers lean toward the idea that the generation of cfDNA is associated with pathological cellular states, which may hinder the application of ni-PGT.

In 2021, the Qiao research team^[48]^ innovatively utilized DNA methylation to investigate the origin of cfDNA in SCM. They conducted targeted DNA methylation sequencing on the culture medium of 194 embryos. The results revealed that 33% of cfDNA in the SCM originated from embryonic cells, 12% from granulosa cells, and 4% from polar bodies. Additionally, the study identified a significant maternal contamination of 33% in the culture medium.

## 5. The reliability of ni-PGT

### 5.1. Reports about the consistency between ni-PGT and PGT

The key issue regarding the widespread clinical application of ni-PGT lies in whether the detection of cfDNA can accurately reflect the genetic status of embryos. Various researchers have provided significantly different results and have shown varying attitudes. Some researchers remain skeptical about the clinical utility of ni-PGT due to inconsistencies between cfDNA results and embryo biopsy findings.^[49]^ While some studies indicate higher consistency in detecting chromosomal aneuploidies with cfDNA compared to traditional TE biopsy, conflicting results have also been reported.

Gianaroli et al^[50]^ found that the concordance rates of ploidy between BF-cfDNA and polar bodies, blastomeres, and TE biopsies were 70.0%, 88.9%, and 82.0%, respectively, in 39 cases using aCGH testing.

Tobler et al’s^[51]^ study revealed a 62% concordance between BF-cfDNA and TE biopsy results in detecting aneuploidy, suggesting caution in utilizing BF for embryo testing.

Magli et al^[52]^ discovered, through aCGH testing, that the concordance rates of ploidy between BF-cfDNA and polar bodies, blastomeres, and TE biopsies were 94%, 95%, and 97.1%, respectively.

Vera-Rodriguez et al^[40]^ observed a 30.4% concordance between aneuploidy detected in SCM-cfDNA from 113 day 3 to day 5 blastocysts using NGS and FISH, and TE biopsies. Additionally, SNP sequencing revealed that SCM-cfDNA is a blend of embryonic and maternal DNA, with embryonic DNA constituting a median percentage of only 8%. In the same year, Ho et al^[53]^ studied 141 embryos, of which only 39% and 80.4% of SCM on the third and fifth day, respectively, successfully amplified cfDNA. On the third day, the concordance rate between SCM-cfDNA testing and WB screening for aneuploidy was 56.3%, while the gender concordance rate was 81.3%. On the fifth day, the concordance rate for aneuploidy between SCM-cfDNA testing and WB screening was 65%, with a gender concordance rate of 70%. On the fifth day, the sensitivity and specificity of SCM-cfDNA testing were 61% and 80%, with negative predictive value and positive predictive value (PPV) at 88% and 47%, respectively.

A study by Rubio et al^[54]^ involving 115 blastocysts found that the concordance rate of SCM-cfDNA in detecting embryonic chromosomal aneuploidies compared to TE biopsy was 78.7%, with a specificity of 71.7% and a sensitivity of 94.5%. In the same year, Huang et al^[55]^ examined the reliability of SCM-cfDNA testing by comparing the results to TE biopsy findings in 52 donated embryos. Their study demonstrated a false negative rate of zero for noninvasive preimplantation genetic testing for aneuploidy (ni-PGT-A) through next-generation sequencing (NGS) analysis, with significantly superior PPV and specificity compared to TE biopsy. Additionally, ni-PGT-A showed higher consistency in detecting chromosome ploidy and chromosome copy numbers than TE biopsy.

In 2020, a significant multicenter randomized controlled trial involving 1301 SCM samples across 4 countries and 8 reproductive centers was conducted by Rubio et al.^[56]^ The study found that the consistency of chromosomal aneuploidy detection between SCM-cfDNA testing and TE biopsy ranged from 72.5% to 86.3%. The SCM-cfDNA testing demonstrated a PPV of 75.0%, a negative predictive value of 83.6%, sensitivity between 76.5% and 91.3%, and specificity from 64.7% to 93.3%.

Cao^[57]^ reported a study on the reliability of ni-PGT for detecting chromosomal mosaicism in embryos. Using WB testing as the gold standard, the study revealed a 100% concordance between SCM and WB testing in detecting chromosomal mosaicism, surpassing the results of TE biopsies. This underscores the advantage of ni-PGT in detecting mosaicism, as cfDNA is derived from the WB, unlike TE biopsies that only sample 5 to 10 cells from the TE. This makes cfDNA a more comprehensive indicator of overall mosaicism in embryos.

In a study by Sethi et al,^[58]^ all samples from 26 embryos undergoing ni-PGT using SCM-cfDNA were successfully amplified for whole-genome sequencing. Through copy number variation analysis, the ploidy concordance rate between SCM-cfDNA and TE biopsy was found to be 68.38%; per chromosome concordance was 85.13% and sex chromosome concordance was 73.0%.

Chow et al^[59]^ found in a study of 153 ni-PGT embryos that the chromosome ploidy concordance rate of SCM and TE could reach 75.4%. They also discovered that by collecting SCM on the 6th day and washing embryos multiple times on the third day, the chromosome ploidy concordance rate was higher. The fertilization method used by ni-PGT is similar in results to IVF when compared to ICSI.

### 5.2. The advantages of SCM-cfDNA over BF-cfDNA

Despite reports of both SCM-cfDNA and BF-cfDNA being utilized in ni-PGT, the prevailing trend in current literature favors the use of SCM over BF for its notable advantages in ni-PGT.

SCM offers convenience in sampling and minimal risk of embryo damage. In contrast, BF is obtained through blastocyst micropuncture, a procedure considered invasive despite studies indicating that the loss of BF does not harm the embryos.^[38]^

The success rate of whole genome amplification (WGA) using BF is reported to range from 34.8%^[60]^ to 87.5%,^[61]^ which is lower than that of SCM. Additionally, the small volume of BF makes sample extraction and transfer challenging, with general volumes of only 1 μL,^[50,51]^ compared to SCM which requires 10 μL or more.^[54]^

## 6. Factors influence cfDNA concentration and measures to increase cfDNA concentration

The reliability of results in ni-PGT using either SCM or BF is significantly hindered by lower cfDNA concentrations. The lower concentration of cfDNA not only leads to amplification failure of cfDNA, but also increases the proportion of maternal contamination. Factors influencing cfDNA content include:

### 6.1. The time for cfDNA collection

The concentration of cfDNA in embryo culture medium varies with the developmental stage. As embryos grow, the total number of embryonic cells increases, leading to a rise in cfDNA concentration in the culture medium. However, artificially prolonging the embryo culture time to enhance cfDNA concentration may impact embryo development and the success rate of embryo transplantation. Therefore, optimizing the collection time to maximize cfDNA concentration without compromising embryo development is a crucial consideration.

Rubio et al^[54]^ reported a higher success rate in detecting SCM samples collected on days 5 and 6 compared to those collected on day 4. In a separate study by Ho et al,^[53]^ which involved 41 normal fertilizations, it was found that SCM samples collected on day 5 showed a higher success rate compared to those collected on day 3 (80.4% vs 39.0%). This suggests an elevated cfDNA content in SCM samples obtained on day 5, in contrast to those obtained on day 3 or day 4.

### 6.2. The volume of the embryo culture medium

During embryo culture, an excessive volume of culture medium droplets can lead to dilution of cfDNA concentration, while smaller droplets may impact embryo development. Optimizing the embryo culture system to achieve the desired cfDNA concentration without compromising embryo development is crucial for successful ni-PGT. In the study by Rubio et al,^[54]^ using 10 μL droplets to culture embryos, the chromosomal ploidy consistency between SCM-cfDNA and TE biopsy results reached 84%. Huang et al^[55]^ reported that culturing blastocysts in 15 μL droplets, using WB sequencing as the gold standard, showed a concordance rate of 93.8% (45/48) for SCM-cfDNA and 82.0% (41/50) for TE biopsy in determining embryo ploidy. In a large-scale study involving 1301 embryos, Rubio et al^[56]^ found that culturing blastocysts in 10 μL of medium until day 6 and using SCM for ni-PGT resulted in higher cfDNA concentration, as well as better blastocyst formation rate and quality compared to other studies reported in the literature.^[62]^ The authors propose that the improved blastocyst formation with a minimal medium volume may be due to the necessity of factors secreted during embryo development for blastocyst formation, which could be amplified by using small-volume culture medium. Nevertheless, additional research is required to explore the potential effects of prolonged exposure to small-volume culture medium on embryo development.^[41]^

### 6.3. The quality of the embryo

Embryonic morphological grading may influence the content of cfDNA, possibly due to the generation of cfDNA being associated with cellular pathological conditions such as aneuploidy, apoptosis, and fragmentation.^[40–42,46,47]^ Embryonic cells with lower morphological scores may release more cell fragments and exhibit a higher likelihood of apoptosis, leading to increased cfDNA levels. Advanced maternal age is associated with a greater occurrence of embryonic aneuploidy in comparison to younger women,^[11]^ as aneuploid cells are usually removed by the embryo’s self-correction mechanism, leading to the generation of cfDNA. Rule et al^[63]^ quantified the concentration of BF and observed a correlation (*R*^2^ = 0.200) between the content of BF-cfDNA and the morphological grading of day 5 blastocysts. In a study involving 800 blastocysts, Stigliani et al^[19]^ observed the release of cfDNA into the culture medium starting from day 2. The concentration of cfDNA increased with age in female patients, with significantly higher levels observed in elderly women compared to younger women. Embryos with low morphological scores exhibit significantly higher levels of cfDNA in the SCM compared to high-quality embryos.

### 6.4. Detection follows the combination of SCM and BF

In order to improve the success rate and consistency of detection, researchers have increased the cfDNA content by blending SCM with BF. This was achieved by inducing blastocyst contraction through laser perforation, allowing the BF to flow out and mix with SCM. Kuznyetsov et al^[64]^ analyzed a mixture of SCM and BF from 19 fresh embryos and 28 frozen embryos. The study found that both fresh and frozen blastocysts had a successful cfDNA amplification rate of 100%. Moreover, the consistency of cfDNA chromosomal aneuploidy results with TE biopsy and WB testing ranged from 87.5% to 100%. Other studies have also shown that after combining SCM and BF, 87.5% to 100.0% of embryonic cfDNA amplification is successful, with concordance rates of 70.4% to 100.0% for aneuploidies compared to WB or TE, and 76.3% to 100.0%.^[43,64–70]^ This indicates the significance of mixing SCM and BF. However, some scholars have found that combining SCM and BF does not enhance detection efficacy.^[71]^ Additionally, the removal of BF may impact cell communication between the blastocyst and its surrounding environment.^[71]^

## 7. Challenges in the research and application of ni-PGT

### 7.1. Maternal and exogenous DNA contamination

Many scholars consider exogenous DNA contamination to be a primary factor influencing the reliability of ni-PGT results. Potential sources of exogenous contamination include residual maternal granulosa cells, DNA released from polar bodies, excess sperm attached to the zona pellucida during fertilization, and environmental contamination during embryo culture.^[41,54,56,72]^ It is worth noting that due to the extremely low levels of cfDNA in the culture medium, even minimal contamination from maternal or exogenous sources can significantly interfere with the test results, leading to misdiagnosis of embryos and the birth of offspring with genetic disorders. Rubio et al^[54]^ observed a discrepancy in which the TE biopsy indicated a female embryo, while the SCM-cfDNA testing revealed a male embryo. This inconsistency may be attributed to external contamination from plastic products and culture media used during the embryo manipulation process.

Most studies on ni-PGT have utilized ICSI to fertilize oocytes, aiming to limit the introduction of additional sperm into the embryo culture medium. The primary source of exogenous DNA contamination may stem from maternal granulosa cells in ni-PGT. Additionally, before fertilization, efforts are made to remove granulosa cells from around the zona pellucida of the oocyte, yet residual granulosa cells may persist.^[73]^ Hammond et al^[71]^ utilized qPCR to detect the presence of the testis-specific protein Y-linked 1 gene (TSPY1) and the TBC1 domain family member 3 gene (TBC1D3) on chromosome 17 in 227 discarded blastocysts’ SCM. Their findings revealed that only 20% of the SCM samples tested positive for the Y chromosome, whereas theoretically, it should be approximately 50%. This apparent gender imbalance could be attributed to maternal granulosa cell contamination in the SCM. Chen et al^[48]^ investigated the impact of maternal contamination in SCM on determining the chromosomal copy number of embryos. The results revealed that the discordance rate and false negative rate of chromosomal copy numbers increased with higher rates of maternal contamination from polar bodies, granulosa cells, and other sources. Consequently, when contamination occurs in SCM, the detected chromosomal copy numbers in embryo cells are rendered inaccurate.

The presence of trace human DNA in embryo culture media is also a significant factor contributing to exogenous DNA contamination. The culture medium used for embryos is typically supplemented with human serum albumin, which may introduce exogenous human DNA and consequently interfere with cfDNA analysis results. Reports suggest that low-copy genomic DNA and mitochondrial DNA can be detected in culture medium that has not been exposed to embryos, possibly originating from human blood components added to the medium.^[40]^ Notably, the quantity of human DNA in the culture medium can vary among different brands of media and within batches of the same brand, resulting in considerable uncertainty.^[74]^ The human DNA present in the culture medium is typically of male genomic origin,^[75]^ which may cause inaccuracies in the testing results of female embryos and could elevate the ratio of chimeric embryos. As ni-PGT technology becomes more widespread, the key to resolving this issue lies in creating embryo culture media that is free from human-derived DNA.

### 7.2. Selection of the correct “gold standard” for ni-PGT

Researchers evaluating the reliability of ni-PGT may utilize various reference standards or “gold standards” based on specific circumstances, including TE cells,^[40,49,53,54,56,66,76–83]^ polar bodies,^[84]^ WB.^[49,53,79,81,85,86]^ Different reference standards may yield disparate outcomes. The most prevalent research utilizes TE cells as a reference standard, enabling a comparison of ni-PGT results without altering the patient’s transplantation process. The consistency between TE biopsy results and the actual chromosomal status of embryos is high.^[87]^ However, TE biopsy cannot eliminate the impact of embryo mosaicism. One limitation of polar body biopsy is its inability to detect chromosomal abnormalities arising during the postfertilization meiotic division of the zygote, as it only reflects errors in oocyte meiotic division. Studies have shown that up to 20% of embryos identified as euploid through polar body biopsy exhibit aneuploidy during the blastocyst stage.^[88]^ Utilizing WB testing can reflect the overall chromosomal status of the embryo, but this can only be done using discarded embryos donated by patients for research purposes. The ideal gold standard for evaluating ni-PGT would be the results of fetal prenatal diagnosis or newborn chromosomal testing. However, it is uncommon to use fetal or newborn chromosomal results as the gold standard, since ni-PGT or PGT involves the selection of embryos with normal chromosomes or low levels of mosaicism for implantation.

### 7.3. The impact of embryo mosaicism

The occurrence of embryo mosaicism presents a common issue for both traditional PGT and ni-PGT. Reported rates of embryo mosaicism exhibit notable discrepancies, ranging from 15% to 90% during the blastomere stage and from 3% to 24% during the blastocyst stage.^[89,90]^ The self-correction mechanism of embryos may lead to the elimination and apoptosis of abnormal cells in mosaic embryos, causing false positive results of chromosomal abnormalities in cfDNA.^[60]^ Consequently, normal embryos may be discarded, underscoring the challenge of accurately assessing embryo abnormalities solely based on the detection of mosaicism in cfDNA testing.

### 7.4. The success rate of cfDNA amplification

Successful WGA of cfDNA is essential for subsequent analysis. Compared to genomic DNA from TE cells, cfDNA has a lower concentration, is fragmented, and is prone to uneven amplification, leading to allele dropout and resulting in genotyping errors.

Various WGA methods, such as multiple displacement amplification (MDA), multiple annealing and looping-based amplification cycles, SurePlex, PicoPlex, and Reproseq, are currently utilized, each affecting the success rate of amplification differently. In a study by Hanson et al,^[80]^ multiple annealing and looping-based amplification cycles were employed to amplify TE biopsy cells and cfDNA from 166 embryos. The amplification success rate for TE biopsy cells was 100% across all 166 embryos, whereas 37.3% (62/166) of cfDNA samples failed to amplify successfully. The accuracy of MDA amplification is high, yet it demands a substantial amount of initial template DNA. Consequently, a decrease in template DNA leads to reduced coverage and accuracy of the amplification products. Shamonki et al^[76]^ employed MDA technology to amplify 57 cfDNA samples, with only 2 meeting the subsequent detection criteria. Overall, there is no consensus on which WGA method is more suitable for amplifying cfDNA.

### 7.5. The standard operating procedure is absent

Due to the low concentration of cfDNA, the embryo-related procedures in ni-PGT differ from the conventional workflow in IVF laboratories. Adjustments such as the volume of culture droplets, removing granulosa cells, timing of cfDNA sampling, and embryo culture protocols need to be tailored around ni-PGT. However, there is currently no universally accepted standard operating procedure for ni-PGT, leading to discrepancies in the reliability of conclusions reported in the literature. While ICSI is commonly utilized for fertilization in numerous studies, there is evidence from multiple research studies indicating that the conventional IVF fertilization method does not influence ni-PGT outcomes.^[46,83]^ In Xie et al’s^[83]^ study, when utilizing SCM of sperm and Zona pellucida for WGA and NGS, it was found that sperm DNA could not be amplified. In this study, 5 μL of lysis buffer was added to 10 μL of SCM to obtain SCM-cfDNA each time. The authors believe that the WGA conditions used in this study were relatively mild, which were unable to amplify sperm DNA.^[83]^ This effectively avoids sperm DNA contamination from IVF. However, this conclusion may not be applicable to other types of WGA.

Assisted hatching is frequently utilized in the majority of ni-PGT studies to facilitate the release of cfDNA.^[49,69,77]^ However, a study by Hanson et al^[80]^ contradicted this approach, demonstrating that assisted hatching does not enhance the release of embryonic DNA or improve result reliability. The detection results of cfDNA can be influenced by different cfDNA detection platforms in various research studies. The primary platforms for genetic testing of cfDNA at present are array comparative genomic hybridization (aCGH), NGS, and SNP chip. Gianaroli et al^[50]^ utilized aCGH technology to compare the concordance of results between BF-cfDNA and TE biopsy, blastomere biopsy, and polar body biopsy, yielding consistency rates of 82.0%, 88.9%, and 70.0%, respectively. Tsuiko et al^[61]^ utilized NGS methodology to compare the concordance of BF-cfDNA with TE biopsies and ICM karyotyping, revealing a consistency rate of only 40%. Various testing platforms yield different results, with NGS technology demonstrating higher accuracy and sensitivity in detecting Mosaicism compared to aCGH.^[91]^

Apart from this, there is a lack of standards for other parameters in ni-PGT procedures, such as culture conditions and whether embryos have undergone freezing, in various research studies. Differences in the inclusion criteria for samples across various studies, such as patient hormone levels, age, medication regimens, among other factors, can impact embryo quality.^[92]^ These variations may indirectly lead to differences in cfDNA content in both BF and SCM, thus influencing detection results.

## 8. Summary and future directions

In conclusion, due to significant variability in the reliability of ni-PGT across studies, as well as unresolved issues related to genetic contamination and the reliability of WGA of cfDNA, ni-PGT currently cannot be established as a standard diagnostic method or replace traditional PGT based on embryo biopsy. Nonetheless, ni-PGT demonstrates substantial potential for clinical applications, suggesting an expanded scope for PGT through further development and widespread adoption. Future research on ni-PGT may focus on developing cfDNA detection methods to distinguish maternal and fetal DNA, optimizing embryo culture and sampling protocols, establishing NGS bioinformatics analysis strategies tailored for ni-PGT, and designing culture media specifically suited for ni-PGT. Additionally, prospective studies with larger sample sizes are needed to further elucidate the impact of ni-PGT on pregnancy outcomes. Given the respective advantages and disadvantages of PGT and ni-PGT, the combined utilization of both represents a novel approach. Additionally, just like pregnant women undergoing PGT should receive prenatal diagnostic testing,^[93,94]^ it is recommended that all pregnant women conceiving through ni-PGT undergo prenatal diagnostic testing as well.

## Author contributions

**Conceptualization:** Shaozhe Yang, Xiuhong Fu.

**Methodology:** Bo Xu.

**Writing—original draft:** Yuan Zhuang, Qingwei Zhang.

**Writing—review & editing:** Junfeng Li.
